# Beef production and the beef evaluation system in Chile: description, characterization, and quality

**DOI:** 10.1093/af/vfae003

**Published:** 2024-04-16

**Authors:** Arias Rodrigo

**Affiliations:** Instituto de Producción Animal, Universidad Austral de Chile, Valdivia, 5110566, Chile; Centro de Investigación de Suelos Volcánicos, Universidad Austral de Chile, Valdivia 5110566, Chile

**Keywords:** Chilean beef systems, conjugated linoleic acids, grass-fed, human health, phytochemicals

**Implications**

- Most Chilean beef production is based on pastoral systems with low-technology inputs.
- Beef from Chilean production systems is rich in conjugated linoleic acid, omega-3, and phytochemicals that are considered healthier, especially those from grasslands with high species diversity.
- Chilean system of classification of cattle and beef classification and nomenclature is mandatory. However, it does not focus on beef quality but on carcass quality.

## Introduction

Chilean beef production systems traditionally have been considered of low technology, particularly the cow-calf segment, generating low profits compared with the dairy sector, crops, and fruticulture. Over the years, the activity has been displaced from central to southern and austral regions. The main reasons are 1) the high competition using agricultural soils in central Chile, turning from animal production systems toward vineyards, fruticulture, crops, and horticulture production, which generate more profits, and 2) the greater availability of grasslands and permanent pastures in southern and austral regions and soils of limited agricultural potential. In Chile, during the last 5 yr, the average of heads slaughtered annually was 800.1 thousand heads of cattle, producing 207.4 thousand tons of beef. By contrast, the average for the period 2002 to 2017 was 831.0 thousand heads slaughtered producing 213.1 tons of beef. The per capita consumption in the country is 23.5 kg/yr, with 52% of the demand imported mostly from MERCOSUR (Common Market of the South) countries such as Brazil and Paraguay. Compared with other meats, beef per capita consumption is lower than pork and poultry (24.1 and 41.2 kg/yr, respectively). Livestock, and particularly beef production, has been declining in Chile during the last two decades for multiple reasons. Not only climate change and land use opportunities for other more profitable crops but also public policies and regulations that have produced a disincentive and loss of competitiveness in the sector. In addition, the unrestricted entry of beef from MERCOSUR is also a cause of this setback.

**Figure 1.**
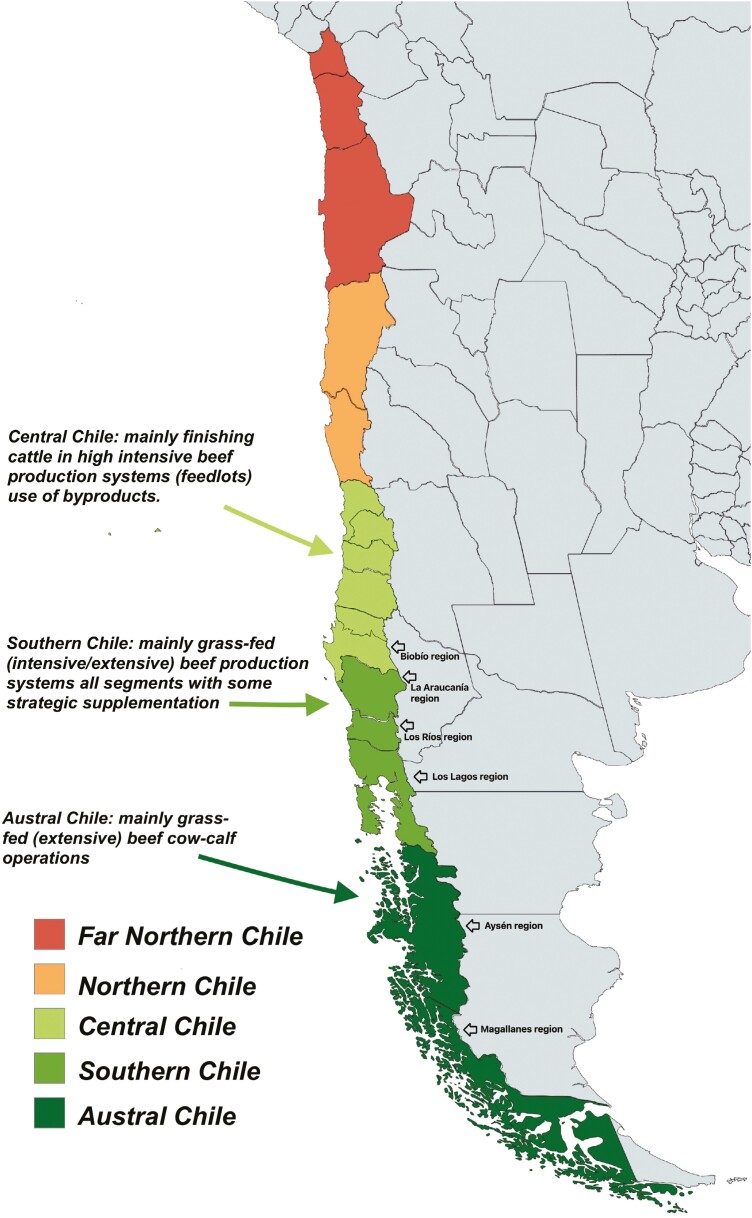
Chilean regional map and its relationship with beef production systems .

In Chile, beef production is mainly done in the southern and austral zones, which include five regions: La Araucanía, Los Ríos, Los Lagos, Aysén, and Magallanes (Figure 1). Together these regions represent more than 54% of the national surface for productive purposes (24.7 million hectares), with a great variety of climates and grasslands. These regions represent 88% of improved pastures, 61.7% of established pastures, and 75.8% of natural grasslands. In Los Ríos and Los Lagos regions, the climate is temperate and rainy, whereas towards the Austral zone (Aysén and Magallanes) cold climates and steppe climates are observed. In all these regions, livestock farming is a relevant activity in economic terms. According to the National Institute of Statistics (INE, 2020), 75.8% of the cattle in the country are concentrated in these five regions. Up to early 2000, beef production in Chile was historically and mainly carried out with dual-purpose breeds (German, Dutch, and Swiss Friesians). However, since then specialized beef breeds have increased their share of the total stock. Currently, according to the latest official figures (INE, 2020), the main breeds for beef production are Angus (28.8%), beef crossbreeds (28.4%), Hereford (5.8%), and other beef breeds (4.2%) the remaining 32.8% correspond to dairy breeds and dairy crossbreeds. Among these, other breeds highlight the French ones such as Charolais, Limousin, Gascon, Basdaiz, and Salers as well as Red Angus, Wagyu, Belgian Blue, Shorthorn, and Highland. Finally, all male calves and a proportion of the females from the dairy sector also contribute a significant proportion to the beef industry. In these pastoral systems, feeding is based on the growth curve of the pastures, which varies depending on the agroecological region and the botanical composition of the grassland. In general, the growth of pastures is quite marked and seasonal, with active and quick growth in the spring, reduced in the summer, a rebound in the autumn, and minimal to no growth in the winter. For this reason, in many production systems, it is essential to have forage conservation to avoid the lack of feed during these critical periods, especially in the winter and eventually in the summer seasons. Being in the southern hemisphere as you move south and further from the equator or closer to the pole and from west to east, there is a delay in both the date at which pastures begin to grow as well as in the maximum production reached, due to climatic conditions and latitude, achieving a lower production peak and less rebound in autumn.

## Productive Cycle

### Cow-calf stage

Cow-calf systems are usually located in areas where soils have a very low to low production potential with animals under continuous grazing and utilize minimum technology and handling. In most of the operations, the calving season is very extensive and does not necessarily match the growth curve of the pastures in the spring. In general cows and heifers have poor nutritional and feeding management affecting their reproductive performance because they are on the marginal lands. Basic infrastructure is nonexistent in most operations or when present in very regular or bad conditions. Since 2013, by law, all cattle in Chile must have a unique Individual Identification Radiofrequency Device (DIIO), an eartag, that allows identification and traceability throughout the whole life of each animal. The oversight of this identification is under the Agricultural and Livestock Chilean Service (SAG), which administrates “The Livestock Information System” (SIPEC) (https://sipecweb.sag.gob.cl/acceso/), and a computer system, which allows the entry, maintenance, and management of data from different technical programs of the Livestock Protection Division, where the updated records on livestock and animal establishments are kept. In this regard, there is a great variation among farmers in the time when calves are tagged. Many of them prefer to do it as soon as possible after birth whereas others do it later or even at the moment of animals leave the farm whether due to the sale or due to transportation to another location (farm) of the same owner.

### Backgrounding stage

In general, weaning occurs at 6 to 7 or even as late as 8 mo of age with live body weight ranging from 220 to 280 kg or even more, depending on the food resources available in each area. Typically, 1 mo before weaning, male calves are castrated, mainly surgically or by burdizzo technique, and then all calves are generally sold in cattle auctions. A lower proportion of calves are sold directly at the ranch directly to the buyer or retained in the ranch for the backgrounding and finishing stages. The backgrounding period varies according to the regions and the resources ranchers have; calves born in the spring are weaned in autumn. Thus, they must cope with the winter in which the calves may have different growth rates or even lose body weight. Consequently, there are numerous and diverse backgrounding systems across the country. The aim is for animals to gain at least 100 to 150 kg of live weight reaching average daily gain (ADG) of 0.7 to 1.2 kg/d in this stage, weighting at the end of this stage 325 to 375 kg of live weight. As mentioned before, animals must cope with the winter when grass growth is minimal or nonexistent depending on the region of the country. The ADG in this period could be less than 0.5 kg/d, and in many operations, there is a loss of body weight due to a lack of food and challenging weather conditions (rain, wind, and mud), which increase the energy required for maintenance purposes. Therefore, cattle are generally supplemented with conserved forage (haylage or silage most commonly). During the following spring, animals experience compensatory gain with ADG up to 1.2 kg/d including some short periods up to 1.8 kg/d, depending on the characteristics of the pasture (botanical composition and digestibility).

### Finishing stage

**Figure 2.**
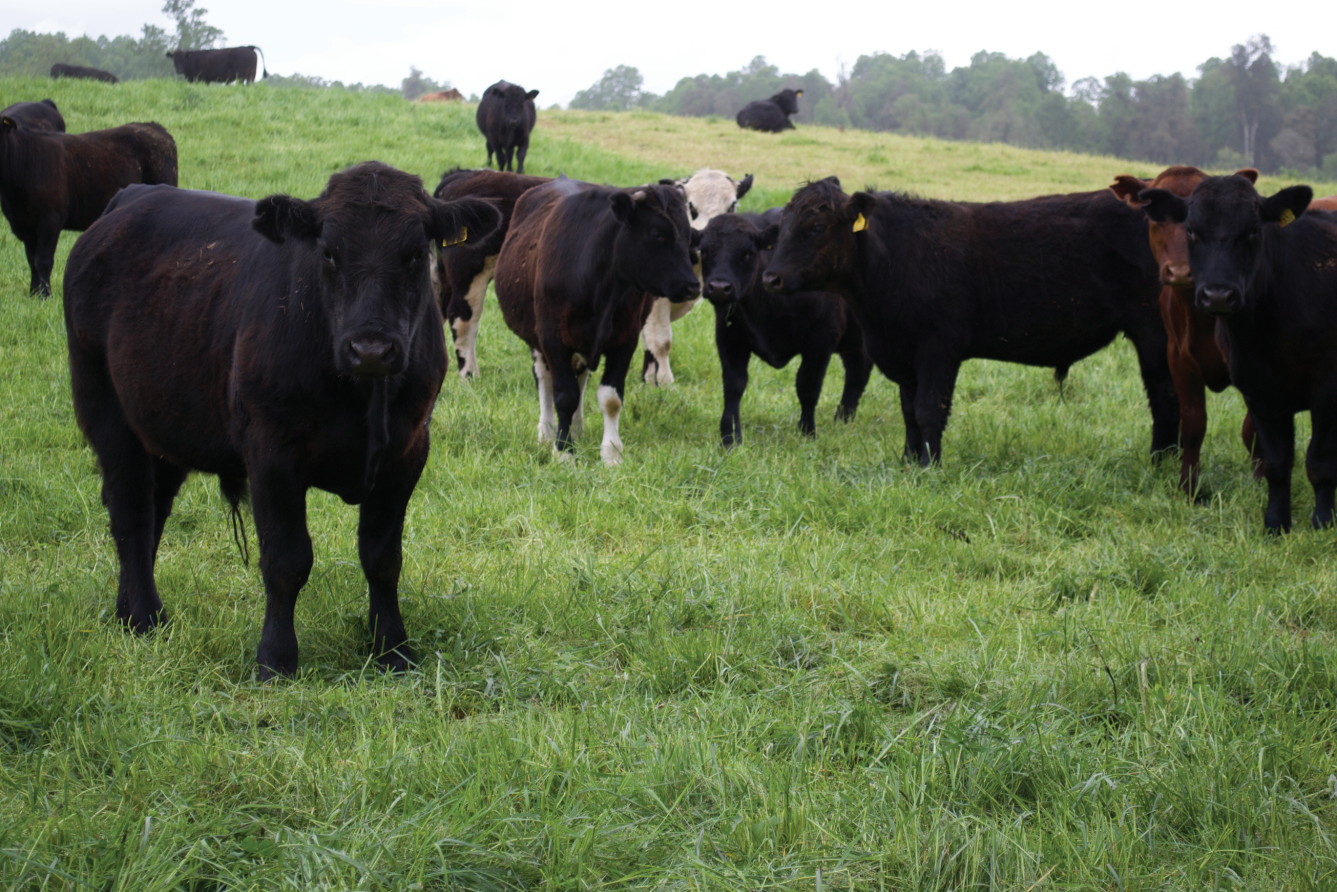
Typical landscape of the Chilean production system in southern regions. Crossbreed steers grazing on perennial pastures during the springtime in the foothills of the “Los Ríos” region.

Finally, the finishing stage may be done in feedlots or on pastures. Usually, steers coming from dairy farms are finished in feedlots using growth-promoting implants, which are allowed in Chile and regulated by the SAG. In the case of beef breeds, they may stay on pasture of good quality (2.65 to 2.95 ME/kg of dry matter , 1.25 to 1.05 NEg/kg of dry matter, 15% to 25% crude protein, 42% to 48% neutral deterrgent fiber) and digestibility, or they may be finished in feedlots with a balanced total mixed ration diet. In the Austral region (Magallanes), the production of calves for slaughter at weights of 310 to 320 kg at 9 to 10 mo of age with late weaning and supplementation (energy) has been also evaluated (Sales et al., 2019). This system is popular in Argentina, and it is known as “bolita calves” producing lighter carcasses with similar or improved beef quality characteristics compared with the traditional finishing of steers on pasture. In Los Ríos and Los Lagos regions, the production of young bulls that are slaughtered at 12 to 14 mo of age with live weights of 380 to 420 kg has been evaluated. However, most producers in the regions of La Araucanía, Los Ríos, Los Lagos, and Aysén finished both steers and heifers (separately) in semi-intensive grazing systems that are slaughtered at 18 to 24 mo of age (Figure 2). These pastures allow an ADG of 1.2 to 1.5 kg/d during the spring allowing the animals to reach final body weights of 450 kg for heifers and 480 to 520 kg for steers. When animals of continental breeds are fattened, they end up with about 50 kg more live weight to achieve the fat coverage demanded by the industry. In these cases, as well as in more extensive systems, the animals take longer, exceeding 24 mo but generally <30 mo old. These animals are heavier, being dual-purpose breeds often less precocious than meat breeds.

## Chilean System of Classification of Cattle and Beef Classification and Nomenclature

It was not until 1992 that Chile established a mandatory system to regulate the beef industry (Law 19,162). This law created a system of classification of livestock and beef classification and nomenclature as well as regulated the operation of slaughterhouses, packing, and establishments in the beef industry (https://bcn.cl/31aw6). To apply the provisions of the Law and achieve its objectives, the Chilean National Institute of Standardization prepared regulations and technical standards that regulate the following areas: 1) beef industry, slaughterhouses, and packing plants; 2) establishments or industries that, in any way or under any circumstance, process, trim or handle beef for wholesale and retail sale; 3) means of transportation for livestock and beef; and 4) packing of beef. The law typifies cattle carcasses according to the class of animal, their dental chronometry, level of fat cover, and bruises (Table 1). The animal classes include 10 groups with the most relevant being steers, heifers, and cows which altogether represent above 97% of slaughtered animals annually. The level of fat cover (FC) includes four categories: FC-0, there is practically no covering fat; FC-1, the covering fat is of scarce thickness but covers most of the carcass; FC-2, abundant fat covering without being excessive, does not form clumps. It covers practically the entire carcass; and FC-3, excessively abundant fat covering with an uneven distribution, presenting certain areas of cumulus clouds. Lastly, the classification structure includes three degrees of bruises: 1) first-degree contusions (affect the subcutaneous tissue, reaching the superficial muscle aponeurosis, causing insignificant injuries there); 2) second-degree bruises (are those that have reached the muscle tissue, injuring it to a greater or lesser depth and extent. It will be observed that the region of the contusion appears hemorrhagic); and 3) third-degree bruises (compromised bone tissue, the muscle tissue generally appears friable with great serous exudation and usually with fracture of the bones in the affected area).

**Table 1.** Current normative for cattle classification and typification of beef carcass in Chile

Category	Class	Dental chronometry/age	Fat coverage
V	Young animals (Heifer, Steers, young bulls)	Maximum 2 permanent teeth	1–2–3
	Young cows and Yearlings	Maximum 4 permanent teeth (up to 30 mo)	
C	Young cows and Steers	Maximum 6 permanent teeth (30 up to 42 mo)	1–2–3
U	Mature Cows	Maximum 8 permanent teeth with Central leveled	1–2–3
	Old cows	From the leveling of permanent medium seconds	
	Oxen	From 4 permanent teeth	
	Bullocks and bulls	From 4 permanent teeth	
N	All classes with exception of calves	No requirements	1–2–3 (Bruises allowed)
O	Calves	No leveling of temporary central incisors	No requirement

Originally, there were six categories representing the word “VACUNO” (cattle in Spanish) in a similar way used by the European Union regulation that uses the word “EUROP”. However, since 2002 the law was modified, eliminating category “A”, and recasting it with “V”. In categories “V,” “C,” and “U,” the fat cover can be 1, 2, or 3. Calves can be included in category “V” if the carcass meets the minimum fat coverage and is at least 160 kg. Another important difference between the European Union regulation, as well as with those from other developed countries, is Chilean producers do not receive economic incentives to produce higher beef quality. In summary, the “V” category corresponds to young animals, such as steers, bulls, and heifers with up to 4 permanent teeth and is usually associated with more tenderness and better beef quality, the “C” category comes from steers and cows up to 3.5 yr old (6 permanent teeth), “U” corresponds to adult cows, oxen, bulls, and young bulls (8 permanent teeth or animals of previous categories with minor bruises), “N” corresponds to all animals that do not meet the requirements of the previous categories, except calves, or those that present higher degree bruises. Finally, “O” includes calves with a maximum of 9 mo of age. The category “N” allows bruises at different levels because is generally dedicated to industrial use.

In many countries, producers are paid based on a grid that rewards better carcass and beef quality (e.g., USA, Australia, and Ireland). Most plants in Europe use a pricing grid that coincides with the EUROP classification grid and sets a base price with bonuses and deductions depending on how the animals are classified. In Australia, young grass-fed cattle that met Meat Standards Australia (MSA), a standard measure index that predicts eating quality and potential merit of a whole carcass calculated using only attributes influenced by preslaughter production, potentially received on average an additional AUS $0.27/kg over-the-hooks compared with non-MSA cattle. Considering the average weight of the carcass (287 kg), it potentially equated to an additional AUS $77.49 per head (MLA, 2021). Currently, a large majority of cattle in Chile are marketed at cattle auctions so producers receive a payment based on age and animal live body weight. Animals with good conformation (e.g., wide and thick back from a rounded shoulder to round buttocks), although not officially considered in payment, are desired and receive better prices. A lower proportion of finished cattle are sold directly to slaughterhouses, where producers receive a payment based mainly on cold carcass weight. Neither of those alternatives rewards carcass quality or even eating quality.

Since Chilean law enforcement, the law has had positive effects such as 1) reduction of slaughterhouses that are not sanitarily suitable for slaughtering animals, whose meat and by-products are intended for human consumption; 2) great progress in slaughterhouse infrastructure and slaughter methods; 3) notable decrease in bruises produced during animal transport and antemortem treatment, due to greater care in animal treatment; 4) reduction of the age of slaughter of the animals; 5) cold chain application; and 6) transparency in retail sales to the public by eliminating the fraud of supplanting a cut of higher value for another of lower value. However, the biggest concerns about the system are 1) the obligation, which is unique in the world; 2) the confusion from the consumers because the concept of carcass quality was mistakenly transferred to beef quality. In this sense, during the last decade, Chilean consumers have been in the process of learning and being educated to distinguish different qualities of beef, which slowly have translated into a gradient of prices based on beef quality; and 3) the application of the Chilean standards to imported beef that currently represents up to 55% of the beef consumed in the country. In fact, many consumers in Chile consider the “V” category to be the best quality and give them a good consumer experience at the sensory level, unaware there are differences in quality within the animal’s carcass cuts. Likewise, in the marketing chain, and particularly in supermarkets, only “V” category beef is sold, which makes it difficult the commercialize the other categories.

It is important to mention the current Chilean Beef Law does not focus on beef quality. In fact, this denied Chile the ability to a preferential beef quota granted by the European Union (No 481/2012) as compensation for its rejection of products from animals with hormones from the United States. The Chilean proposal was rejected by the EU because the current typification did not contain quality parameters such as the evaluation of the maturity by bones and cartilage ossification; color and texture characteristics of the *longissimus dorsi* muscle; evaluation of probable palatability characteristics based on the specific characteristics of intramuscular fat (marbling), the firmness of the *longissimus dorsi* muscle and pH of the same muscle. In addition, animals must be <30 mo, which in the 100 d before slaughter, at least, have only been fed with rations consisting of no <62% of cereal concentrates or by-products (dry matter basis), and whose ME content up to 2.93 Mcal/kg DM (12.26 MJ/kg DM). These animals must be fed with these rations in amounts equal to or greater than 1.4% of their body weight. Therefore, in 2013, the Chilean parliament approved a norm that complies with the provisions contained in Annex I of Regulation No. 620/2009 of the European Union (Nº 5309/2013; SAG, 2013) which established requirements in beef production to provide certification of beef quality of superior quality. This norm considered the parameters associated with beef quality (fat and muscle color, marbling, and physiology maturity), which are the same as included in Annex I of the regulation of the European Union. Nevertheless, since then it has been never applied and Chile never has had access to that beef quota.

One of the biggest concerns of beef industry actors associated with the law arose after the country began increasing beef imports. In many instances, the category assigned to these imports is not the same as what the same piece of beef would have been assigned by Chilean standards. This means the imported beef from old animals (cows, bulls, and oxen) can be categorized as “V,” impacting prices that favor importers, mainly supermarket chains. Thus, one of the main criticisms is whether the beef that comes from abroad is really what they say since it is very difficult to verify the information recorded in the imported beef is true. Thus, a modification of the law has been discussed for several years, proposing that imported meats should use the letter “I” (for imported) in addition to having the flag of the country of origin. The main goal is to make the information transparent, so the consumer is clear about the origin of the product. Additionally, this amendment could allow the national meat, of high quality, to be differentiated from imported one.

Chilean beef law only applies to cattle and not to other species, generating an asymmetry with other meats, and showing an inequity in the trade requirements. The same legislation requires that beef cuts be labeled and marketed according to the carcass category (“VCUNO”) from which they come. This is extensible to cuts of imported origins that want to enter the Chilean market introducing another factor that is impossible to control because is outside of Chile’s frontiers. In addition, the association of the “V” category with a higher quality, which is not necessarily true, detracts from the value of animals in other categories making their marketing difficult. It is well known that techniques such as beef aging can improve quality and consumer eating experience. Likewise, it allows fraud and prevents feedback from the consumers to the producers. The lack of control of imported beef makes it easier for beef of different origins and qualities to be mixed in the trade, hindering a growing demand for a brand associated with quality. Therefore, the Chilean beef law favors traders but not consumers resulting in an incentive to transgress the law.

## Chilean Beef Quality and Chilean Consumer’s Preferences

Predominant Chilean beef production systems are associated with a lower environmental impact and less stress on the animal (IICA, 2004), topics that are of great concern to consumers worldwide (Realini et al., 2023). Currently, there is a great interest in beef pastoral production systems, particularly in those with botanically diverse pastures, due to the benefits associated with the environment, animal welfare, and health as well as human health (Arias et al., 2022; Kearns et al., 2023).

Chilean consumers value beef from animals raised in grazing systems with higher expectations and preferences for beef produced from pastures compared to that produced by animals fed with grains (Schnettler et al., 2008). Nevertheless, there is a minimum level of intramuscular fat to satisfy these higher demands and expectations (Morales et al., 2013a). It is well known the animal’s diet has an important effect on beef’s fatty acid profile. Diverse studies indicate animals fed on pastures have beef with a darker color (Poveda-Arteaga et al., 2023), leaner and less infiltrated fat, and a lower percentage of monounsaturated fatty acids in the subcutaneous fat (Noci et al., 2007). Likewise, the ω-6:ω-3 ratio decreases while the ω-3 and α-linoleic fatty acids increase (Aurousseau et al., 2007). In addition to the nutritional effect, it has been observed the flavor and texture of the beef, as well as the acceptability of the consumers, are also affected by the differences in the composition of the fatty acids (Font i Furnols et al., 2009), with beef with moderate intramuscular fat being more preferred by Chilean consumers.

Beef produced in Chile is very lean with a fatty acids profile considered healthy (Table 2) independent of the finishing system. It is important to mention the Chilean feedlot systems are quite different from those found in the United States, Argentina, Australia, or Canada because in the Chilean case, the diet includes a higher proportion of fiber. In fact, at least 50% of diet corresponds to roughage, usually in the form of silage or haylage in comparison to 7% to 12% of roughage in the Northern Hemisphere feed yards. The values of intramuscular fat found in Chilean beef are similar to those reported on European and Uruguayan beef from animals of 2 to 3 yr of age (De la Fuente et al., 2009) fed on grasslands (1.76% to 2.36%) but lower than those reported for pasture + supplementation (2.92% to 2.95%) and also those reported in Argentina (Latimori et al., 2008). Larraín and Vargas-Bello (2013) point out that Chilean beef could be classified as extra lean according to the USDA guidelines (<4.5% of total fat), except for ribeye, which would enter the lean category (<10% of fat). Contreras (2005) assessed the effect of the type of supplement on steers of the Red Chilean Friesian breed reporting a diet with a greater amount of grain supplement increased live and carcass weight, without affecting beef quality attributes. Other authors reported oat grain supplementation (2.5 kg/head/d for 101 d) decreases the ω-3 fatty acid content and increases the ω-6:ω-3 ratio of steers slaughtered at 380 kg live weight (Klee et al., 2011). Similar results were reported by Catrileo et al. (2014) in dairy bulls, where bulls fed grazing and concentrate at 1% of live body weight, showed a lower ω-6:ω-3 ratio than those fed with a 2% diet based on grain (approximately 7 kg/animal/d).

**Table 2.** Intramuscular fat and relative composition of fatty acids (%) of the *longissimus thoracis* muscle (Adapted from Morales et al., 2012).

Variable	Finishing diet (30 d) Grass-fed (*n* = 80)	Grass-fed + supplementation^*^ (*n* = 94)	Chilean Feedlot (*n* = 46)
Intramuscular fat (%)	2.33	2.29	2.20
Rumenic acid (CLA)	0.88^a^	0.70^b^	0.61^c^
Saturated fatty acids	0.88^a^	0.70^b^	0.61^c^
Monounsaturated fatty acids	49.34^b^	49.61^b^	52.30^a^
Polyunsaturated fatty acids	43.29^a^	42.53^a^	39.78^b^
PUFA:SFA	5.13	5.65	5.71
ω-6 fatty acids	3.13^b^	3.54^b^	4.03^a^
ω-3 fatty acids	1.92^ab^	2.08^a^	1.63^b^
ω-6: ω-3 ratio	1.75^b^	1.84^b^	2.79^a^

^*^Supplements are usually small cereal grains; the daily amount is 0.3% to 1.0% of live body weight. Different superscripts represent statistical differences between columns at 5% significance.

Morales et al. (2013b) compared the effects of two biotypes (Angus × Hereford crossbred and Holstein Friesian) on beef quality in Chile. Steers were fed under a pastoral system plus supplementation during wintertime (silage, hay, and concentrate). It should be noted the grassland consisted mainly of *Lolium perenne* L. (45%), *Holcus lanatus* L. (24%), and *Bromus valdivianus* Phil. (13%). The experiment ended in January when the availability and quality of pasture began to decline. The dairy breed steers weighed between 500 and 510 kg and beef breeds between 450 and 470 kg. There were no differences between both biotypes regarding chemical composition, pH, and fatty acid profile. In addition, these values were like those reported in the literature. The authors concluded that beef from dairy breed steers had greater tenderness and marbling than the beef crossbreed, showing a different raw meat color detected in a trained sensory panel and instrumentally. Nevertheless, both groups were considered beef of great tenderness and good quality. Similar results were reported by Morales et al. (2023) for Holstein steers in Uruguay who concluded that increasing pasture in the diet changes the color of muscle and fat, increasing conjugated linoleic acid (CLA) content.

Pastoral beef such as that produced in southern Chile contains different CLA isomers that are considered beneficial for human health, highlighting its antiatherogenic and antithrombogenic effects (Dilzer and Park, 2011). On the other hand, vaccenic (*trans*-11 C18:1) and rumenic (*cis*-9 *trans*-11 C18:2) acids reduce insulin resistance (Nguyen et al., 2019). It has been suggested vaccenic acid (*trans*-11 C18:1) attenuates dyslipidemia, fatty liver, and inflammation (Jacome-Sosa et al., 2016), by reducing pro-inflammatory cytokines and platelet aggregation (Jaudszus et al., 2012). For their part, Gebauer et al. (2011) point out that rumenic acid (*cis*-9 *trans*-11 C18:2) reduces the risk of cardiovascular diseases and cancer, increases bone mass, and modulates the immune and inflammatory response. It is important to note the concentrations of these acids are higher in animals fed in grazing systems than in animals fed with concentrates. On the other hand, recent research indicates cattle grazing on diverse plant-species grasslands concentrate a wider variety and higher amounts of phytochemicals in beef compared to grazing monoculture pastures, while phytochemicals are further reduced or absent in beef of grain-fed animals (van Vliet et al., 2021, 2023; Kearns et al., 2023). Animal fats from pastoral systems present a much lower value of ω-6 as well as the ω-6: ω-3 ratio of 4:1 recommendation by the WHO. Thus, Chilean beef from the different production systems meets these recommendations (Table 2). In addition, pasture-finishing broadly improves animal metabolic health and accumulates additional potential health-promoting phytochemical compounds in their meat compared to concentrate-finishing diets (grain-fed) as reported by van Vliet et al. (2023). These authors found >1,500 unique compounds in beef, having revealed previously unrecognized differences in animal metabolic health and nutritional composition because of finishing mode. Whether observed nutritional differences have an appreciable effect of these phytochemicals on human health remains to be determined, due to beef being a complex matrix food. At present, a study is ongoing comparing 2 typical Chilean production systems to assess the profile of phytochemical compounds showing beef from a pastoral system has 10 times more flavonoid glycosides, 4 times more carboxylic acids, and 3 times more flavonoids than those finished in a conventional Chilean feedlot. Likewise, beef has 2 times more contents of hydroxybenzoic acids, hydroxycinnamic acids, phenolic alcohol, and flavanols (data not published). Some of these phytochemicals (terpenoids, phenols, carotenoids, and antioxidants) found in meat and milk in amounts comparable to those found in plant foods, which are known to have anti-inflammatory, anticancer, and cardioprotective effects (van Vliet et al., 2021). In this sense, the ongoing paradigm shift in human nutrition can represent an important opportunity for pastoral production systems due to the benefits of these production systems in terms of quality and nutrition, animal welfare, and environmental impact (Arias et al., 2022).

## Conclusion

In conclusion, there is a Chilean system for beef assessment but it does not reward producers for the quality of beef they produce, which added to the lack of knowledge of the population regarding beef quality and has led to consumer confusion. Consequently, producers do not have an economic stimulus to reward their efforts, creating a vicious cycle. On the other hand, there are multiple productive agroecosystems in the country, most of which could improve beef production and further highlight the sustainability and quality of the beef produced. Finally, there is a lack of national public–private policy to promote production, research, and outreach to ensure food security and consumer education in the country.

## About the Authors

**Rodrigo Arias** is an Associate Professor at the Institute of Animal Production, Faculty of Agricultural and Food Sciences at the Universidad Austral de Chile (UACh). He is also the Director of the Doctorate Program in Agricultural Science and is a member of the Council of the School of Agronomy. He is an active member of the Chilean Society of Animal Production and leads the Livestock, Health, and Sustainability group. He obtained the degree of University Technician in Agricultural Production from Pontificia Universidad Católica de Chile and his Bachelor´s degree from Universidad Católica de Temuco. He earned both his M.Sc. and Ph.D. in Animal Science (ruminant nutrition) with a minor in Statistics, from the University of Nebraska-Lincoln (USA). His research focuses on the relationship between climatic factors, nutrition, and the quality of products (beef and milk) and their effect on people’s health. He also studies the effects of grass-fed production systems on nitrogen balance and enteric methane emissions. He has led and collaborated in projects funded by Fondecyt, FIC, UACh-Virginia Tech, CONICYT, Global Research Alliance, and Norman E. Borlaug International Agricultural Science and Technology Fellowship Program, among others. **Corresponding author:**rodrigo.arias@uach.cl
